# Risk and timing of postpartum depression in parents of twins compared to parents of singletons

**DOI:** 10.1111/acps.13766

**Published:** 2024-10-25

**Authors:** Sofie Egsgaard, Mette Bliddal, Lars Christian Lund, Simone N. Vigod, Trine Munk‐Olsen

**Affiliations:** ^1^ Research Unit of Child and Adolescent Psychiatry, Department of Clinical Research University of Southern Denmark Odense Denmark; ^2^ Clinical Pharmacology, Pharmacy and Environmental Medicine, Department of Public Health University of Southern Denmark Odense Denmark; ^3^ Research unit OPEN, Department of Clinical Research University of Southern Denmark Odense Denmark; ^4^ Women's College Hospital and Research Institute University of Toronto Toronto Ontario Canada

**Keywords:** mental health, multiple birth, parents, postpartum depression, twin birth

## Abstract

**Background:**

Parents of twins appear to be at increased risk of postpartum depression (PPD), yet little is known about the magnitude and timing of onset in the postpartum period compared to singleton parents.

**Methods:**

We conducted a cohort study using the Danish nationwide health registers. We defined a study population of parents that is, mothers and fathers of all twin and singleton livebirths between 1997 and 2019. Postpartum depression was defined as incident depression diagnosis or a redeemed antidepressant prescription from childbirth through 365 days postpartum. We performed a parametric time‐to‐event analysis based on Poisson regression. The time scale was time since birth, modeled using restricted cubic splines. From this we estimated the hazard ratio (HR) representing the momentary risk, and the cumulative risk ratio (RR) over the first year postpartum, in twin compared to singleton parents.

**Results:**

The study population was based on 27,095 twin and 1,350,046 singleton births. In adjusted analyses, the HR of twins compared to singletons was highest around 2 months postpartum (HR 1.28, 95% CI 1.10–1.49) for mothers, and around 6 months (1.20, 95% CI 1.02–1.42) for fathers. The 6 months adjusted cumulative RR of PPD in twins compared to singletons was 1.24 (95% CI 1.10–1.40) for mothers and 1.11 (95% CI 0.95–1.30) for fathers.

**Conclusions:**

Twin mothers had increased risk of PPD compared to singleton mothers, which was driven by an immediate increase after childbirth. The risk among twin fathers was not increased immediately after childbirth, but we found slightly elevated risk around 6 months postpartum. This could suggest diverse patterns of PPD symptomatology in twin parents compared to singleton parents and between mothers and fathers. Our findings underline parents of twins as a potentially vulnerable group to PPD and emphasize the need for increased awareness of their mental health.

**Significant outcomes**

- Mothers of twins have an elevated risk of postpartum depression compared to singleton mothers, which is especially pronounced in the early postpartum period and persists until about 6 months postpartum.
- Fathers of twins have indication of a slightly increased risk of postpartum depression around 6 months postpartum compared to fathers of singletons.

**Limitations**

- We used antidepressant prescriptions and depression diagnoses to measure postpartum depression, which may only capture moderate to severe cases of postpartum depression.
- Our findings could be vulnerable to detection bias if twin parents are more likely to have recognized postpartum depression.
- A delay between PPD onset and time of registered PPD could likely affect our observed ratios over time, meaning that the true peaks were in fact earlier than observed.

## 1 INTRODUCTION

Postpartum depression (PPD) is the most common mental disorder after birth with a prevalence of 10% to 15% among mothers^1^, ^2^ and 8% to 9% among fathers.^3^, ^4^ The causes of PPD most likely include a combination of biological changes during pregnancy to postpartum,^5^, ^6^ factors related to delivery,^7^, ^8^ stressors of parenthood,^9^ or heritable factors.^10^

Approximately 2% of childbirths are twins.^11^ The incidence of twin births has risen due to both increasing maternal age and use of assisted reproductive technologies (ART), though recently the impact from ART on twin pregnancies has decreased.^12^ Becoming a parent of twins has been linked to increased PPD risk,^13^, ^14^, ^15^ but the evidence on this topic is limited and primarily based on small study samples, particularly regarding fathers.

Different factors could explain why twin parents would have increased PPD risk. Twin pregnancies are at increased risk of obstetrical complications, such as preterm birth and cesarean sections, which are associated with PPD.^16^, ^17^ Further, caring for two infants instead of one could increase stressors of parenthood in the postpartum period.^16^, ^17^ If PPD in twin parents is linked to some of the specific conditions related to delivering and parenting twins, the trajectories and time of PPD onset in the postpartum period could also be different from those of singleton parents.

However, most previous studies have only measured PPD at one point postpartum, limiting the ability to describe the temporality of PPD onset between twin and singleton parents following childbirth. Improving our understanding of the temporality of PPD onset in twin parents could potentially guide the development of targeted prevention and interventions supports and services for this group and provide clues to underlying causes of PPD.

## 2 AIM

The aim of the study was to investigate (i) the risk of PPD in parents of twins compared to singletons and (ii) compare timing of PPD onset across the first year postpartum.

## 3 MATERIALS AND METHODS

We conducted a register‐based cohort study investigating the risk of PPD following the first year after childbirth among mothers and fathers of twins compared to mothers and fathers of singletons from 1997 to 2019.

### 3.1 Data sources

The Danish health registers served as data platform, in which linkage between registers is possible using a unique personal identifier.^18^ The Civil Registration System holds information on vital status, sex, and birthdate on all individuals alive and living in Denmark since 1968.^19^ The Danish Medical Birth Register (Birth Register) contains information on all children born in Denmark and their parents, pregnancy‐ and birth related factors – including multiple gestations – since 1973.^20^ The Danish National Register of Assisted Reproductive Technology (ART Register) provides data on In Vitro Fertilization (IVF) and Intracytoplasmic Sperm Injection (ISCI) treatment in public and private Danish fertility clinics since 1994.^21^ From 2006 it also included information on Intrauterine insemination (IUI) treatments.^21^ The Danish National Patient Register (Patient Register) contains information on all psychiatric inpatient and outpatient diagnoses given at hospital facilities since 1995.^22^ The Danish National Prescription Register holds data on all redeemed prescription medication using the WHO Anatomic Therapeutic Classification (ATC) system and date of dispensing since 1995.^23^

### 3.2 Study population

Our study population was formed based on all singleton and twin pregnancies resulting in livebirths between January 1, 1997, and December 31, 2019, which were identified from the Birth Register. Twin gestations including a stillbirth, triplet‐, and higher‐order gestations were excluded. From eligible childbirths we defined two populations of parents: We defined a population of *mothers*, which in this and the following refers to the gestational parent who was registered as carrying the pregnancy in the Birth Register. We defined a population off *fathers* referring to the registered parent in the Birth Register who did not carry the pregnancy. We defined this group regardless of sex (it was possible for same‐sex couples to have a person of female sex registered) as we aimed to investigate the impact on the non‐birthing parent. However, we kept the terminology “father” as we expected the vast majority to be of male sex. We used a two‐year washout period to describe only new onset of depressive episodes postpartum and thus excluded a parent who had any filled antidepressant prescription (ATC code N06A) or a recorded depression diagnosis (ICD‐10 codes F32‐33) within the 2 years prior to childbirth. Note, applying the washout period may give different number of included mothers and fathers to the study population.

### 3.3 Exposure

The exposure was becoming a parent (e.g., mother or father) of liveborn twins versus becoming a parent of a liveborn singleton. Therefore, the exposure start date was the date of childbirth.

### 3.4 Outcome and follow‐up

The outcome of interest was PPD, which was defined as the first of either a recorded primary or secondary depression diagnosis (ICD‐10 code F32‐33) or a redeemed antidepressant prescription (ATC code N06A) at any time from the date of childbirth to 365 days (1 year) postpartum (Figure S1). Depression diagnosis was considered a proxy for severe depression, as this could only be diagnosed from a hospital contact, whereas antidepressant prescriptions was considered as a proxy for moderate to severe depression, since this could also be prescribed from general practitioner and therefore reflect depression treated in primary care only.^24^ To increase specificity of depression case status and include only the more severe cases of PPD, we repeated all analyses defining outcome by diagnoses only.

### 3.5 Covariates

We obtained information on the following variables for adjustment and descriptive purposes: Information on parental age at childbirth was obtained using the Civil Register. We defined a livebirth as obtained from ART‐treatment if the mother had a record of achieved pregnancy in the ART Register, which could be linked to a livebirth in the Birth Register (for elaboration see Table S1).^25^ We defined a parent as cohabiting if living with a father at the date of childbirth. Further, information on parity of mother, neonatal intensive care unit admissions, pregnancy‐ and obstetrical complications was obtained from the Birth Register and the Patient Register (Table S1).

### 3.6 Statistical analysis

We reported descriptive statistics on mothers and fathers and on obstetrical and pregnancy variables for the total number of included twin and singleton childbirths.

All the following analyses were done separately for mothers and fathers:

We performed a parametric time‐to‐event analysis based on Poisson regression.^26^ The primary time scale was time since childbirth split into 30‐day intervals, from which we modeled incidence rates of PPD using a restricted cubic spline with four knots.^27^ In the unadjusted analyses we included interaction terms between exposure (twin or singleton parent) and the time scale. Covariates in the adjusted analyses were identified using Directed Acyclic Graphs and included variables that were assumed to be a cause of the outcome or on a backdoor path (Figure S2). We included parental age, calendar year (both included as restricted cubic splines), ART‐treatment, and cohabitation status. We included interaction terms between ART‐treatment and calendar year to account for decreasing number of twin pregnancies arising from ART over time.^28^ For mothers, we further included parity (primiparous or multiparous) (Figure S2). We did not include pregnancy‐ and obstetrical complications as covariates, as we defined these as intermediates, that is, a part of the causal pathway of being pregnant with and becoming parents of twins. All analyses were clustered to account for parents who were included to the study with more than one childbirth.

Using this approach, we modeled observed and adjusted incidence and cumulative incidence rates. Adjusted rates were obtained from standardizing the modeled rates in twins and singletons with fixed covariate values, and rates were standardized based on the marginal distribution (i.e., mean values) of the included covariates in the study population. We visualized both observed and adjusted cumulative incidence rates per 1000 persons in twin and singleton parents. Based on the modeled incidence rates, we estimated unadjusted and adjusted hazard ratios (HR) representing the momentary risk of PPD in twin compared to singleton parents at any specific time between childbirth and 365 days postpartum. The purpose of the HR was to identify specific time points in which twin parents were at a potentially increased risk compared to singleton parents. Based on cumulative incidence rates we also estimated risk ratios (RR) representing the cumulative risk of PPD in twin compared to singleton parents from childbirth and at 3, 6, 9 and 12 months postpartum. The purpose of the risk ratio was to describe the total risk of PPD in twin compared to singleton parents after the given time points postpartum.

As a sensitivity analysis we repeated our main analysis in which we only included parents of children born between 2007 and 2019, in which period data to be used for adjustment from the ART register was considered more complete. Further, we conducted supplementary analyses in which we repeated the main analysis separately for depression diagnoses and antidepressant prescriptions as outcome measures for PPD. We further reported descriptive statistics on number of PPD cases obtained from diagnoses and prescriptions, respectively. All analyses were conducted using *R* Studio version 4.3.2, including package *epi* for statistical modeling approach.^29^

#### 3.6.1 Ethics

The study was registered at The University of Southern Denmark (registration no. 11.627) and approved by the Danish Health Data Authorities. Because of the legislation in Denmark, Register‐based studies do not require ethical approval or informed consent. The study's protocol was pre‐registered and is available at https://osf.io/c5zrb.

## 4 RESULTS

We identified 25,611 twin mothers and 1,257,947 singleton mothers (in total 746,623 unique mothers) and correspondingly 25,457 twin and 1,271,646 singleton fathers (in total 745,967 unique fathers) (Table 1). Twin parents were slightly older, more likely to be primiparous and cohabiting compared to singleton parents. Twin births were to a higher extent obtained from ART treatment and had substantial more pregnancy‐ and obstetrical complications and neonatal intensive care unit admissions than singleton births (Table 1).

**TABLE 1:** Characteristics of the study population.

	Twin	Singleton
**Mothers, *n***	**25,611**	**1,257,947**
Age, median (IQR^a^)	31.0 (28.0, 35.0)	30.0 (27.0, 33.0)
Calendar year, *n* (%)		
1997–2004	9796 (38.2)	469,981 (37.4)
2003–2011	8547 (33.4)	381,914 (30.4)
2012–2019	7268 (28.4)	406,052 (32.3)
Primiparous, *n* (%)	12,585 (49.8)	544,695 (43.8)
Cohabiting, *n* (%)	24,045 (95.5)	1,164,142 (93.4)
**Fathers, *n***	**25,457**	**1,271,646**
Age, median (IQR^a^)	33.0 (30.0, 37.0)	32.0 (29.0, 36.0)
Calendar year, *n* (%)		
1997–2004	9740 (38.3)	471,729 (37.1)
2003–2011	8554 (33.6)	387,038 (30.4)
2012–2019	7163 (28.1)	412,879 (32.5)
Male sex^b^, *n* (%)	25,294 (99.4)	1,268,781 (99.8)
Cohabiting, *n* (%)	24,356 (95.7)	1,192,265 (93.8)
**Childbirths** ^c^ **, *n* (%)**	**27,095**	**1,350,046**
ART‐treatment, *n* (%)	9110 (33.6)	59,733 (4.4)
Preterm birth, *n* (%)	12,932 (48.2)	75,376 (5.6)
C‐section, *n* (%)	14,096 (52.0)	244,290 (18.1)
Preeclampsia/eclampsia, *n* (%)	2672 (9.9)	45,176 (3.3)
Gestational diabetes, *n* (%)	1030 (3.8)	33,638 (2.5)
Postpartum hemorrhage, *n* (%)	3196 (11.8)	81,048 (6.0)
Neonatal intensive care unit admission, *n* (%)	11,695 (43.2)	109,218 (8.1)

*Note*: 15,882 mothers had missing information on parity, 12,514 mothers had missing information on cohabitation (no registered father in the Birth Register) and 12,669 childbirths had missing information on preterm birth.

^a^ IQR = interquartile range.

^b^ Fathers also include individuals of female sex registered as father in the birth register.

^c^ Based on unique number of childbirths included to the study population.

**FIGURE 1:**
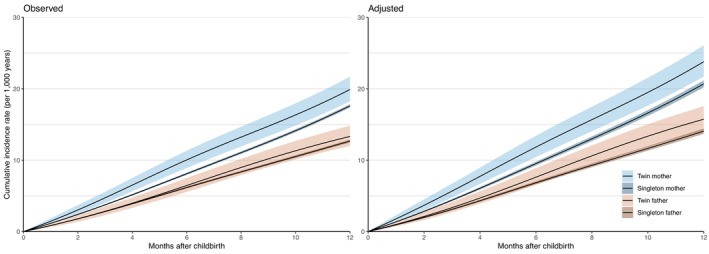
Observed and adjusted cumulative incidence rates of postpartum depression in twin and singleton mothers and fathers from childbirth and 12 months postpartum. Adjusted rates were based on the marginal distribution (i.e., mean values) of the included covariates in the study population. Blue curves represent a mother (of twins or singletons) aged 30 years that gave birth in 2008 with values on cohabitation, ART‐treatment and primiparity of 0.93, 0.05, and 0.44, respectively. Red curves represent a father (of twins or singletons) aged 33, who became a parent in 2007, with values on cohabitation and ART‐treatment of 0.94 and 0.05 respectively.

Figure 1 shows observed and adjusted cumulative incidence rates from time of birth and through 12 months postpartum. The adjusted cumulative incidence rate per 1000 persons at 12 months postpartum was 23.8 (95% confidence interval (CI) 21.7–26.1) for twin mothers, 20.7 (95% CI 20.2–21.2) for singleton mothers and correspondingly 15.7 (95% CI 14.1–17.6) for twin fathers and 14.1 (95% CI 13.7–14.4) for singleton fathers (Figure 1, Table S2).

**FIGURE 2:**
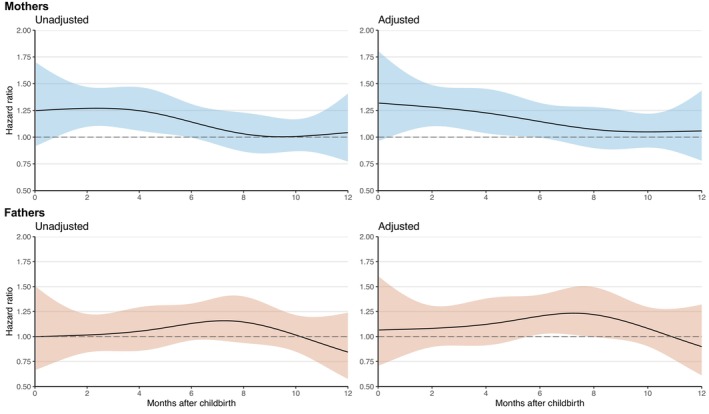
Unadjusted and adjusted hazard ratio of postpartum depression in twins compared to singleton mothers and twin compared to singleton fathers from childbirth and 12 months postpartum. For mothers adjusted for age at childbirth, calendar year, ART‐treatment, cohabitation, and parity. For fathers adjusted for age at childbirth, ART‐treatment, calendar year, and cohabitation.

Figure 2 shows the unadjusted and adjusted HR (the specific risk at a given time postpartum) of PPD in twins compared to singletons for mothers and fathers. For mothers, the adjusted HR was 1.28 (95% CI 1.10–1.49) at 2 months postpartum and declined to 1.15 (95% CI 0.99–1.32) at 6 months and 1.05 (95% CI 0.90–1.22) at 10 months postpartum (Figure 2, Table S3). For fathers, the adjusted HR increased from 1.08 (95% CI 0.89–1.31) at 2 months postpartum to 1.20 (95% CI 1.02–1.42) at 6 months and declined to 1.08 (95% CI 0.90–1.30) at 10 months postpartum (Figure 2, Table S3).

Table 2 shows unadjusted and adjusted RRs (cumulative risk since childbirth) in twin compared to singleton mothers and fathers. The adjusted RR at 3 months postpartum was 1.29 (95% CI 1.09–1.53) for mothers and 1.08 (95% CI 0.87–1.34) for fathers. At 12 months postpartum, the RR was 1.15 (95% CI 1.05–1.26) for mothers and 1.12 (95% CI 1.00–1.25) for fathers (Table 2).

**TABLE 2:** Risk ratio (cumulative risk since childbirth) of postpartum depression among twin compared to singleton mothers and fathers at 3, 6, 9, and 12 months postpartum.

	Mothers			
	Postpartum depression cases (*n*)		Risk ratio (95% CI)	
Time after birth	Twin	Singleton	Unadjusted	Adjusted^a^
3 months	117	4823	1.26 (1.07–1.49)	1.29 (1.09–1.53)
6 months	258	10,131	1.24 (1.10–1.40)	1.24 (1.09–1.41)
9 months	374	15,708	1.18 (1.06–1.30)	1.19 (1.07–1.32)
12 months	505	21,960	1.13 (1.03–1.23)	1.15 (1.05–1.26)

	Fathers			
	Postpartum depression cases (*n*)		Risk ratio (95% CI)	
Time after birth	Twin	Singleton	Unadjusted	Adjusted^b^
3 months	71	3660	1.01 (0.82–1.25)	1.08 (0.87–1.34)
6 months	162	7747	1.05 (0.90–1.22)	1.11 (0.95–1.30)
9 months	262	11,909	1.08 (0.96–1.22)	1.15 (1.01–1.30)
12 months	337	16,016	1.05 (0.94–1.17)	1.12 (1.00–1.25)

^a^ Adjusted for age at childbirth, calendar year, ART‐treatment, cohabitation, and parity.

^b^ Adjusted for age at childbirth, calendar year, ART‐treatment, and cohabitation.

Sensitivity analyses including only parents of children born between 2007 and 2019 showed similar tendencies to the main analysis but had wider confidence intervals (Figure S3, Table S4). Sensitivity analyses using only depression diagnoses as indicator of PPD showed attenuated RR and HR compared to the main analysis and had larger confidence intervals due to fewer cases (Figure S3, Figure S4, Table S4, Table S5). Sensitivity analyses using only antidepressant prescriptions as outcome measure for PPD were similar to the main analysis but had more elevated estimates of RR and HR (Figure S3, Table S4).

## 5 DISCUSSION

### 5.1 Main findings

In this study, we found elevated risks of PPD in twin compared to singleton parents. The elevated risk was driven by an increased risk immediately after childbirth and within the first 6 months postpartum among twin mothers. After 6 months, we observed no differences, as the risk resembled that of singleton mothers. For fathers, we – in contrast – found risk of PPD among twin compared to singletons was not higher in the first period after childbirth but was slightly elevated around 6 months after childbirth.

### 5.2 Comparison to previous studies

Previous research has shown increased risk of PPD in twin compared to singleton mothers,^13^, ^14^, ^15^, ^30^, ^31^ although some have also shown no difference.^13^, ^32^ In the present study, we used a register‐based approach, securing a large study population of both mothers and fathers, and our findings align with a recent population‐based study, which found an increased risk of mental illness in twin mothers compared to singleton mothers from birth till 365 days postpartum.^15^ Most evidence has only measured PPD at one time postpartum, and is therefore unable to map out differences in timing of PPD onset between twin and singleton parents. However, one study that measured PPD at one and 6 months postpartum found that PPD risk in twin mothers was not increased at 1 month postpartum but was increased after 6 months.^14^ This contrasts our findings, as we found PPD risk in twin mothers to be increased immediately after childbirth, but not after 6 months. However, this study used symptoms scores to measure PPD, whereas we used diagnoses and prescriptions, which may represent different levels of PPD severity.

Very few studies have investigated PPD risk in fathers, and have indicated elevated risk of PPD in twin compared to singleton fathers.^13^ This is in line with findings from our study indicating that twin fathers had slightly elevated risk of PPD compared to singleton fathers.

### 5.3 Implications

Our findings suggested that twin mothers were at increased risk of PPD compared to singleton mothers in the first period after childbirth until around 6 months postpartum, after which the relative increase attenuated. Although speculative, several factors could explain an increased risk observed during this time: First, taking care of two infants instead of one may pose a bigger burden and increased stressors in twin mothers compared to singleton mothers.^13^ Second, twin pregnancy and twin birth is associated with increased risk of pregnancy‐ and obstetrical complications as well as neonatal admissions, all of which are related to PPD risk.^7^ It could also be possible that the increased risk in twin mothers is, in part, driven by increased depression in pregnancy that is due to pregnancy complications, but which is only captured after childbirth. Third, if the biological drop in reproductive hormones from pregnant to postpartum that is also in part expected to trigger PPD is higher in twin mothers, this could also make them more susceptible to developing depression.^33^ It should be emphasized that it is also possible that the increased risk in twin mothers could be due to detection bias if these mothers are followed more closely through universal care programs than singleton mothers. However, in Denmark, guidelines for women expecting twins are mainly focused on increased support and follow‐up during pregnancy, whereas no specific recommendations or national practice exist in relation to the postpartum period.^34^, ^35^ Thus, regardless of the potential underlying pathways that could drive the observed associations, our findings point to a window in time, where increased awareness and extra efforts could be done to support mothers of twins.

Contrary to mothers, our findings among fathers indicated an elevated risk of PPD around 6 months postpartum, but not in the first months after childbirth. Whereas fathers are not directly impacted by pregnancy and childbirth as mothers, they are exposed to stressors and burdens from taking care of two infants instead of one. Additionally, the burden of supporting a potentially exhausted mother may further strain the father over time. Although speculative, this could suggest that the impact from such stressors would trigger PPD onset later in the postpartum period. It is also possible that fathers have a delay in detection and recognition of PPD compared to mothers. For instance, there may be less awareness of PPD in fathers, and fathers tend to have different help‐seeking behaviors than mothers,^36^ which could also explain an observed risk increase at a later point. Further, it should be emphasized that the effect sizes for fathers are relatively small, and therefore interpretations and implications are vulnerable to potential biases.

In our findings for both mothers and fathers, our sensitivity analyses indicated more elevated risks when using antidepressant prescriptions as a measure for PPD and attenuated risks when using depression diagnoses. Since depression diagnoses given at hospital facilities are considered more severe marker of depression than antidepressant prescriptions, which may also represent treatment in primary care only, these findings could underline that the increased risk in twin parents mostly apply for less severe depressive episodes. However, since antidepressants are also prescribed for other disorders such as anxiety, it is also possible that these findings represent an increased risk of anxiety or other mental disorders in twin compared to singleton parents.

Although both twin mothers and fathers appeared to be at an increased risk of developing PPD, it should be mentioned that with twin pregnancies births being relatively rare, the added number of PPD cases from parenting twins would be small. Nevertheless, our findings document that twin parents constitute a group that could benefit from increased awareness with respect to their mental health.

### 5.4 Methodological considerations

Strengths of our study include the use of health registers, which ensured a large study population including all mothers and fathers over the entire study period. Further, the data on timing of PPD onset allowed us to describe varying PPD risk after childbirth and not only at one time.

However, the following limitations should be acknowledged:

First, PPD was measured from depression diagnoses and antidepressant prescriptions capturing parents who sought and received help. Therefore, we may only detect moderate to severe PPD cases, and thus fail to capture PPD cases that were not treated or treated with non‐pharmacological treatment in primary care only.^24^ Further, it should be noted that antidepressant prescriptions may be prescribed on other indications and thus capture other disorders than depression. However, for mothers, is has been shown that most antidepressant prescriptions in the perinatal period are for depression treatment, suggesting that it is a suitable proxy for a depression diagnosis.^37^

Second, when considering timing of PPD, it is likely that there is a delay between true PPD onset and the time of registered PPD (depression diagnosis or redeemed prescription). This could affect the observed ratios over time, meaning that the true peaks in risk were in fact earlier than observed. While we do not believe this delay would differ between exposure groups (twin and singleton), it could be more pronounced in our findings among fathers than among mothers, if fathers are less likely to be detected with PPD. Third, our study included PPD cases between childbirth and until 1 year postpartum, whereas PPD may also be defined as depressive episodes occurring only within three or 6 months postpartum.^2^ We included a longer period to detect variations in risk between twins and singleton parents over time, and our analytical approach allowed for us to describe relative risk of PPD at various time points, reflecting both estimates for shorter and longer definitions of the postpartum period. Also, we reported risk of PPD over time by modeling rates with restricted cubic splines. Thus, it should be recognized that this approach is sensitive to the number and placing of knots, which brings uncertainties to the findings. Fourth, we did not have access to information on socioeconomic status, such as education, income level or ethnic background. Even though we do not consider them confounders requiring adjustment after accounting for ART‐treatment (Figure S2), we were not able to compare socioeconomic information between exposure groups. However, if present we hypothesize twin parents would tend to be at higher socioeconomic position than singletons, which would bias the association downwards. Among fathers, we also included individuals with female sex, but did not adjust for sex, since almost all individuals in this group (>99%) were of male sex, and we do not believe this to have inferred our findings. Fifth, the study population is only representative of the Danish population, and generalizability to other populations may be limited.

Finally, it should be noted that the observed cumulative incidence rates of PPD between mothers and fathers may not be comparable in terms of reflecting true disease incidence. Mothers may be less willing to take antidepressant medication postpartum because of uncertainties related to breastfeeding,^38^, ^39^ and therefore antidepressants as markers of depression could reflect different disease severities between mothers and fathers.

## 6 CONCLUDE

In this study we described the risk of PPD among parents of twins compared to parents of singletons, and the timing of PPD onset over the first year after childbirth. We found an increased risk of PPD among twin compared to singleton mothers from childbirth and till approximately 6 months postpartum, but not from 6 months and onwards. Several factors could potentially drive this finding including an increased burden with infant care for two children, increased risk of pregnancy‐and obstetrical complications, but also more awareness among twin mothers. For fathers, we did not observe an increased risk in twins compared to singletons immediately after childbirth but in contrast found indications for a slightly elevated risk around 6 months after childbirth. This could indicate that the increased impact from becoming fathers of twins has onset at a later point postpartum compared to singletons but could also be related to a delay in detection and recognition of PPD compared to mothers. Our findings emphasize parents of twins as a potentially vulnerable group to PPD and emphasize a need for increased awareness in this group, particularly in the immediate time after childbirth and extending into the subsequent months postpartum.

## FUNDING INFORMATION

SE, TMO, and MB are funded by The Novo Nordisk Foundation (grant number NNF21OC0072397). SE has also received funding from The Psychiatric Research Fund in the Region of Southern Denmark (grant number A5752) and The Region of Southern Denmark (grant number A1784). Remaining authors: None.

## CONFLICT OF INTEREST STATEMENT

TMO has received a speaker honorarium from Lundbeck A/S. Remaining authors: None.

### PEER REVIEW

The peer review history for this article is available at https://www.webofscience.com/api/gateway/wos/peer-review/10.1111/acps.13766.

## Supporting information


**Data S1.** Supporting Information.

## Data Availability

This study uses individual‐level register data, which is confidential and cannot be shared according to Danish regulations. Information on application for data access is available at https://sundhedsdatastyrelsen.dk/da/forskerservice/ansog-om-data.
