# Supplementary material for: Risk and timing of postpartum depression in parents of twins compared to parents of singletons
Source: Acta Psychiatr Scand. 2024 Oct 25;151(2):163–72. doi: 10.1111/acps.13766 (PMC11695093; doi:10.1111/acps.13766)
Supplement: Supplementary file 1 — Data S1. Supporting Information. [file ACPS-151-163-s001.docx]

Tables

**Table S1: Definition of covariates obtained from the Danish Medical Birth Register and The Danish National Register of Assisted Reproductive Technology**

| **The Medical Birth Register** | |  |  |
| --- | --- | --- | --- |
| **Variable** | **ICD-10 Code** | **Register ascertainment window (relative to birthdate)** | **Comment** |
| Preeclampsia/eclampsia | O14, DO11, DO24 | [- GA at delivery: +7] | Diagnosis code mother |
| Gestational diabetes | O244 | [- GA at delivery: +7] | Diagnosis code mother |
| Cesaeran section | O82, O842, O843, O843D | [-7 : +7] | Diagnosis code mother |
| Postpartum hemorrage | O720 (+) VPH* | [-7 : +7] | Diagnosis code mother. Supplemented w. supplementary code (+) VPH500 |
| Neonatal care admission | P | [0 : +1] | Diagnosis code child. Supplemented w. hospital unit specialty 080 ("Pædiatri") (English: Pediatric) |
| **The Danish National Register of Assisted Reproductive Technology** | | |  |
| **Variable** | **Definition** |  | **Comment** |
| ART-childbirth | Record of achieved pregnancy in the ART register which could be linked to a livebirth in the MBR which fulfilled that: (i) the duration between ART treatment date and date of childbirth (the assumed duration of pregnancy) did not exceed 308 days and (ii) if this duration did not differ more than 30 days from registered gestational age at birth. | | In case of several eligible treatment dates in which the pregnancy could had occurred, the last one was chosen, and for childbirths with missing information on gestational age (~1%) we considered the birth obtained from ART-treatment if the assumed pregnancy duration was between 140 and 308 days |

**Table S2: Observed and Adjusted cumulative incidence rates of postpartum depression in twin compared to singleton mothers and Fathers from 1 to 12 months postpartum.**

|  |  | | Cumulative incidence per 1000 persons (95% CI) | | | | |
| --- | --- | --- | --- | --- | --- | --- | --- |
|  |  | Mothers | | | Fathers | | |
| Time after birth | model | | Singleton | Twin | Singleton | Twin | |
| 2 months | Observed | | 2.4 (2.3-2.5) | 3.0 (2.5-3.7) | 1.8 (1.7-1.9) | 1.8 (1.4-2.3) |  |
| 4 months | Observed | | 5.2 (5.0-5.3) | 6.5 (5.7-7.5) | 3.9 (3.8-4.0) | 4.0 (3.3-4.8) |  |
| 6 months | Observed | | 8.1 (7.9-8.3) | 10.0 (8.9-11.3) | 6.2 (6.0-6.3) | 6.5 (5.6-7.5) |  |
| 8 months | Observed | | 11.1 (10.9-11.2) | 13.2 (11.9-14.7) | 8.4 (8.2-8.5) | 9.0 (7.9-10.2) |  |
| 10 months | Observed | | 14.1 (13.9-14.3) | 16.3 (14.9-18.0) | 10.5 (10.3-10.7) | 11.3 (10.1-12.7) |  |
| 12 months | Observed | | 17.6 (17.4-17.8) | 19.9 (18.2-21.7) | 12.7 (12.5-12.9) | 13.3 (12.0-14.8) |  |
| 2 months | Adjusted | | 2.8 (2.7-2.9) | 3.7 (3.0-4.5) | 2.0 (1.9-2.1) | 2.1 (1.6-2.8) |  |
| 4 months | Adjusted | | 6.1 (5.9-6.3) | 7.7 (6.7-8.9) | 4.3 (4.2-4.5) | 4.7 (3.9-5.6) |  |
| 6 months | Adjusted | | 9.5 (9.3-9.8) | 11.8 (10.4-13.4) | 6.8 (6.6-7.0) | 7.6 (6.5-8.9) |  |
| 8 months | Adjusted | | 13.0 (12.7-13.3) | 15.7 (14.1-17.5) | 9.3 (9.0-9.5) | 10.6 (9.3-12.1) |  |
| 10 months | Adjusted | | 16.6 (16.2-17.0) | 19.5 (17.6-21.5) | 11.6 (11.3-12.0) | 13.4 (11.9-15.0) |  |
| 12 months | Adjusted | | 20.7 (20.2-21.2) | 23.8 (21.7-26.1) | 14.1 (13.7-14.4) | 15.7 (14.1-17.6) |  |
| Note: Adjusted cumulative incidences of mothers represent a woman aged 30 years that gave birth in 2008 with values on cohabitation, ART-treatment and primiparity of 0.93, 0.05 and 0.44, respectively. Adjusted cumulative incidence of a fathers represent a partner aged 33, who became a parent in 2007, with values on cohabitation and ART-treatment of 0.94 and 0.05 respectively. | | | | | | |  |

**Table S3: Hazard ratio of postpartum depression of twin compared to singleton mothers and fathers from 1 to 12 months postpartum**

|  | Risk ratio (95% CI) | | | |
| --- | --- | --- | --- | --- |
|  | Mothers | | Fathers | |
| Time after birth | Unadjusted | Adjusted^a^ | Unadjusted | Adjusted^b^ |
| 1 months | 1.26 (1.02-1.55) | 1.30 (1.05-1.61) | 1.01 (0.77-1.32) | 1.01 (0.77-1.32) |
| 2 months | 1.27 (1.10-1.47) | 1.28 (1.10-1.49) | 1.02 (0.84-1.23) | 1.02 (0.84-1.23) |
| 3 months | 1.27 (1.10-1.47) | 1.26 (1.08-1.46) | 1.03 (0.85-1.24) | 1.03 (0.85-1.24) |
| 4 months | 1.25 (1.06-1.47) | 1.23 (1.04-1.45) | 1.05 (0.86-1.29) | 1.05 (0.86-1.29) |
| 5 months | 1.20 (1.03-1.40) | 1.19 (1.01-1.40) | 1.09 (0.90-1.31) | 1.09 (0.90-1.31) |
| 6 months | 1.14 (1.00-1.31) | 1.15 (0.99-1.32) | 1.13 (0.96-1.33) | 1.13 (0.96-1.33) |
| 7 months | 1.08 (0.93-1.25) | 1.10 (0.95-1.29) | 1.16 (0.96-1.39) | 1.16 (0.96-1.39) |
| 8 months | 1.03 (0.87-1.23) | 1.07 (0.90-1.28) | 1.15 (0.94-1.40) | 1.15 (0.94-1.40) |
| 9 months | 1.01 (0.85-1.19) | 1.05 (0.89-1.25) | 1.09 (0.91-1.31) | 1.09 (0.91-1.31) |
| 10 months | 1.01 (0.87-1.17) | 1.05 (0.90-1.22) | 1.02 (0.85-1.22) | 1.02 (0.85-1.22) |
| 11 months | 1.02 (0.84-1.24) | 1.05 (0.87-1.28) | 0.93 (0.72-1.20) | 0.93 (0.72-1.20) |
| 12 months | 1.04 (0.77-1.41) | 1.06 (0.78-1.44) | 0.84 (0.57-1.24) | 0.84 (0.57-1.24) |
| ^a^Adjusted for age at childbirth, calendar year, ART-treatment, cohabitation, and parity. ^b^Adjusted for age at childbirth, ART-treatment, calendar year and cohabitation. | | | | |
|  | | | | |

**Table S4 Sensitivity analyses risk ratio and hazard ratios of postpartum depression in twin compared to singleton mothers and fathers. Sensitivity analyses include a) restricting PPD to only depression diagnoses b) restricting PPD to only antidepressant prescriptions and c) restricting study period to only including parents of children born 2007-2019**

|  |  |  | Estimate (95% CI) | | | |
| --- | --- | --- | --- | --- | --- | --- |
|  |  |  | Mothers | | Fathers | |
| Sensitivity analysis | Measure | Time point | Unadjusted | Adjusted^a^ | Unadjusted | Adjusted^a^ |
| a) Diagnoses only | Hazard Ratio | 1 months | 0.91 (0.55-1.51) | 0.94 (0.57-1.57) | 0.89 (0.24-3.33) | 1.12 (0.30-4.20) |
|  |  | 2 months | 1.27 (0.84-1.91) | 1.29 (0.85-1.97) | 0.82 (0.33-2.06) | 1.03 (0.41-2.60) |
|  |  | 3 months | 1.47 (0.92-2.34) | 1.49 (0.92-2.41) | 0.80 (0.32-1.97) | 1.00 (0.41-2.49) |
|  |  | 4 months | 1.41 (0.93-2.12) | 1.43 (0.94-2.19) | 0.85 (0.32-2.27) | 1.07 (0.40-2.87) |
|  |  | 5 months | 1.23 (0.81-1.87) | 1.26 (0.82-1.94) | 1.03 (0.43-2.46) | 1.30 (0.54-3.11) |
|  |  | 6 months | 1.09 (0.66-1.81) | 1.13 (0.67-1.89) | 1.34 (0.67-2.69) | 1.68 (0.84-3.40) |
|  |  | 7 months | 1.03 (0.60-1.76) | 1.06 (0.61-1.83) | 1.60 (0.75-3.40) | 2.02 (0.95-4.31) |
|  |  | 8 months | 1.02 (0.62-1.69) | 1.05 (0.63-1.74) | 1.54 (0.68-3.47) | 1.93 (0.85-4.39) |
|  |  | 9 months | 1.06 (0.67-1.68) | 1.07 (0.67-1.72) | 1.13 (0.51-2.53) | 1.43 (0.64-3.20) |
|  |  | 10 months | 1.13 (0.68-1.88) | 1.13 (0.66-1.91) | 0.69 (0.23-2.07) | 0.87 (0.29-2.62) |
|  |  | 11 months | 1.23 (0.61-2.45) | 1.20 (0.58-2.46) | 0.38 (0.06-2.21) | 0.47 (0.08-2.79) |
|  |  | 12 months | 1.34 (0.52-3.45) | 1.29 (0.48-3.44) | 0.20 (0.01-2.68) | 0.25 (0.02-3.38) |
|  | Risk ratio | 3 months | 1.04 (0.70-1.54) | 1.06 (0.71-1.60) | 0.86 (0.31-2.34) | 1.08 (0.39-2.98) |
|  |  | 6 months | 1.14 (0.84-1.56) | 1.17 (0.84-1.62) | 0.93 (0.44-1.96) | 1.17 (0.54-2.50) |
|  |  | 9 months | 1.12 (0.84-1.49) | 1.15 (0.85-1.56) | 1.13 (0.68-1.88) | 1.42 (0.83-2.41) |
|  |  | 12 months | 1.13 (0.87-1.47) | 1.15 (0.87-1.53) | 0.99 (0.60-1.62) | 1.24 (0.74-2.08) |
| b)  Antidepres-sant prescriptions only | Hazard ratio | 1 months | 1.41 (1.13-1.77) | 1.46 (1.16-1.83) | 1.01 (0.76-1.34) | 1.07 (0.81-1.42) |
|  |  | 2 months | 1.34 (1.14-1.57) | 1.35 (1.15-1.59) | 1.02 (0.84-1.24) | 1.08 (0.89-1.31) |
|  |  | 3 months | 1.27 (1.09-1.48) | 1.25 (1.07-1.47) | 1.03 (0.85-1.25) | 1.09 (0.90-1.32) |
|  |  | 4 months | 1.21 (1.02-1.44) | 1.18 (0.98-1.41) | 1.05 (0.85-1.30) | 1.12 (0.90-1.38) |
|  |  | 5 months | 1.16 (0.98-1.37) | 1.14 (0.95-1.35) | 1.09 (0.90-1.32) | 1.15 (0.95-1.40) |
|  |  | 6 months | 1.12 (0.97-1.29) | 1.12 (0.96-1.30) | 1.13 (0.95-1.33) | 1.19 (1.01-1.41) |
|  |  | 7 months | 1.08 (0.93-1.26) | 1.11 (0.95-1.30) | 1.15 (0.95-1.39) | 1.22 (1.01-1.47) |
|  |  | 8 months | 1.06 (0.88-1.27) | 1.10 (0.92-1.33) | 1.14 (0.93-1.41) | 1.21 (0.98-1.49) |
|  |  | 9 months | 1.03 (0.86-1.23) | 1.09 (0.91-1.30) | 1.10 (0.91-1.33) | 1.16 (0.96-1.40) |
|  |  | 10 months | 1.01 (0.87-1.18) | 1.06 (0.91-1.24) | 1.03 (0.86-1.24) | 1.09 (0.91-1.31) |
|  |  | 11 months | 1.00 (0.82-1.22) | 1.03 (0.84-1.26) | 0.96 (0.74-1.24) | 1.01 (0.78-1.31) |
|  |  | 12 months | 0.98 (0.72-1.35) | 0.99 (0.72-1.37) | 0.88 (0.60-1.30) | 0.93 (0.63-1.37) |
|  | Risk ratio | 3 months | 1.37 (1.15-1.64) | 1.40 (1.16-1.68) | 1.02 (0.82-1.26) | 1.08 (0.86-1.34) |
|  |  | 6 months | 1.26 (1.11-1.44) | 1.26 (1.10-1.45) | 1.05 (0.90-1.22) | 1.11 (0.95-1.30) |
|  |  | 9 months | 1.19 (1.07-1.32) | 1.20 (1.08-1.35) | 1.08 (0.95-1.22) | 1.14 (1.00-1.30) |
|  |  | 12 months | 1.14 (1.03-1.25) | 1.15 (1.04-1.28) | 1.06 (0.95-1.18) | 1.12 (1.00-1.25) |
| c)  Study period from  2007-2019 | Hazard ratio | 1 months | 1.22 (0.93-1.60) | 1.28 (0.97-1.69) | 1.01 (0.71-1.44) | 1.08 (0.76-1.54) |
|  |  | 2 months | 1.34 (1.11-1.62) | 1.37 (1.13-1.66) | 0.98 (0.77-1.26) | 1.05 (0.82-1.35) |
|  |  | 3 months | 1.43 (1.19-1.72) | 1.43 (1.19-1.74) | 0.97 (0.75-1.25) | 1.03 (0.80-1.33) |
|  |  | 4 months | 1.45 (1.18-1.78) | 1.44 (1.16-1.78) | 0.97 (0.74-1.29) | 1.04 (0.79-1.38) |
|  |  | 5 months | 1.37 (1.13-1.67) | 1.36 (1.11-1.67) | 1.01 (0.78-1.31) | 1.08 (0.84-1.40) |
|  |  | 6 months | 1.22 (1.02-1.47) | 1.23 (1.02-1.48) | 1.08 (0.86-1.35) | 1.15 (0.92-1.44) |
|  |  | 7 months | 1.07 (0.87-1.32) | 1.09 (0.88-1.35) | 1.14 (0.89-1.46) | 1.22 (0.95-1.57) |
|  |  | 8 months | 0.95 (0.74-1.21) | 0.98 (0.76-1.26) | 1.17 (0.89-1.54) | 1.25 (0.96-1.65) |
|  |  | 9 months | 0.89 (0.70-1.13) | 0.93 (0.73-1.19) | 1.16 (0.91-1.48) | 1.24 (0.97-1.58) |
|  |  | 10 months | 0.88 (0.71-1.09) | 0.93 (0.76-1.16) | 1.11 (0.88-1.40) | 1.19 (0.94-1.50) |
|  |  | 11 months | 0.91 (0.70-1.19) | 0.97 (0.74-1.27) | 1.05 (0.75-1.45) | 1.12 (0.81-1.55) |
|  |  | 12 months | 0.95 (0.63-1.46) | 1.01 (0.66-1.55) | 0.98 (0.60-1.60) | 1.05 (0.64-1.71) |
|  | Risk ratio | 3 months | 1.28 (1.04-1.58) | 1.33 (1.07-1.65) | 1.00 (0.75-1.32) | 1.07 (0.80-1.42) |
|  |  | 6 months | 1.34 (1.15-1.56) | 1.36 (1.15-1.60) | 1.00 (0.82-1.23) | 1.07 (0.87-1.32) |
|  |  | 9 months | 1.23 (1.08-1.40) | 1.25 (1.09-1.44) | 1.05 (0.89-1.24) | 1.12 (0.94-1.33) |
|  |  | 12 months | 1.14 (1.01-1.28) | 1.17 (1.03-1.33) | 1.06 (0.91-1.22) | 1.13 (0.97-1.32) |
| ^a^Adjusted for age at childbirth, calendar year, ART-treatment, cohabitation, and parity. ^b^Adjusted for age at childbirth, ART-treatment, calendar year, cohabitation, and maternal age. | | | | | | |

|  | Mothers  (n = 1,283,558) | Fathers  (n = 1,297,103) |
| --- | --- | --- |
| Depression diagnosis or antidepressant prescription (%) | 22,465 (1.75) | 16,353 (1.26) |
| Depression diagnosis (%) | 2575 (0.20) | 825 (0.06) |
| Antidepressant prescriptions (%) | 20,153 (1.57) | 15,589 (1.20) |
| Depression diagnoses only (%) | 2312 (0.18) | 764 (0.06) |
| Antidepressant prescriptions only (%) | 19,890 (1.55) | 15,528 (1.20) |
| Both diagnosis and prescription (%) | 263 (0.02) | 61 (0.00) |

**Table S5: Number of PPD cases among mothers and fathers by depression measure (depression diagnoses and antidepressant prescriptions) and overlap between measures**

**Figures**

**Figure S1: Individual level entry of study cohort**

**
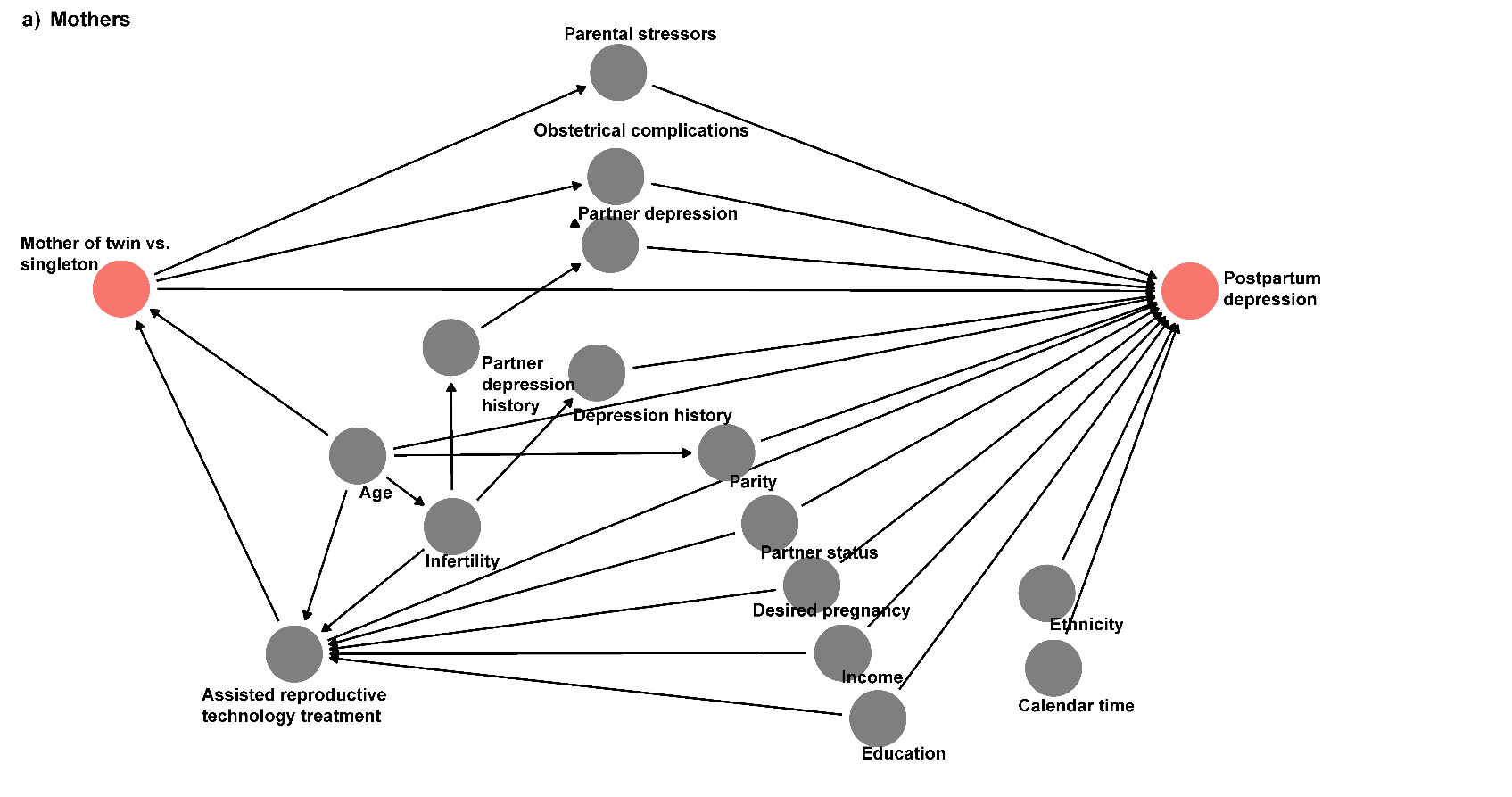
**

**
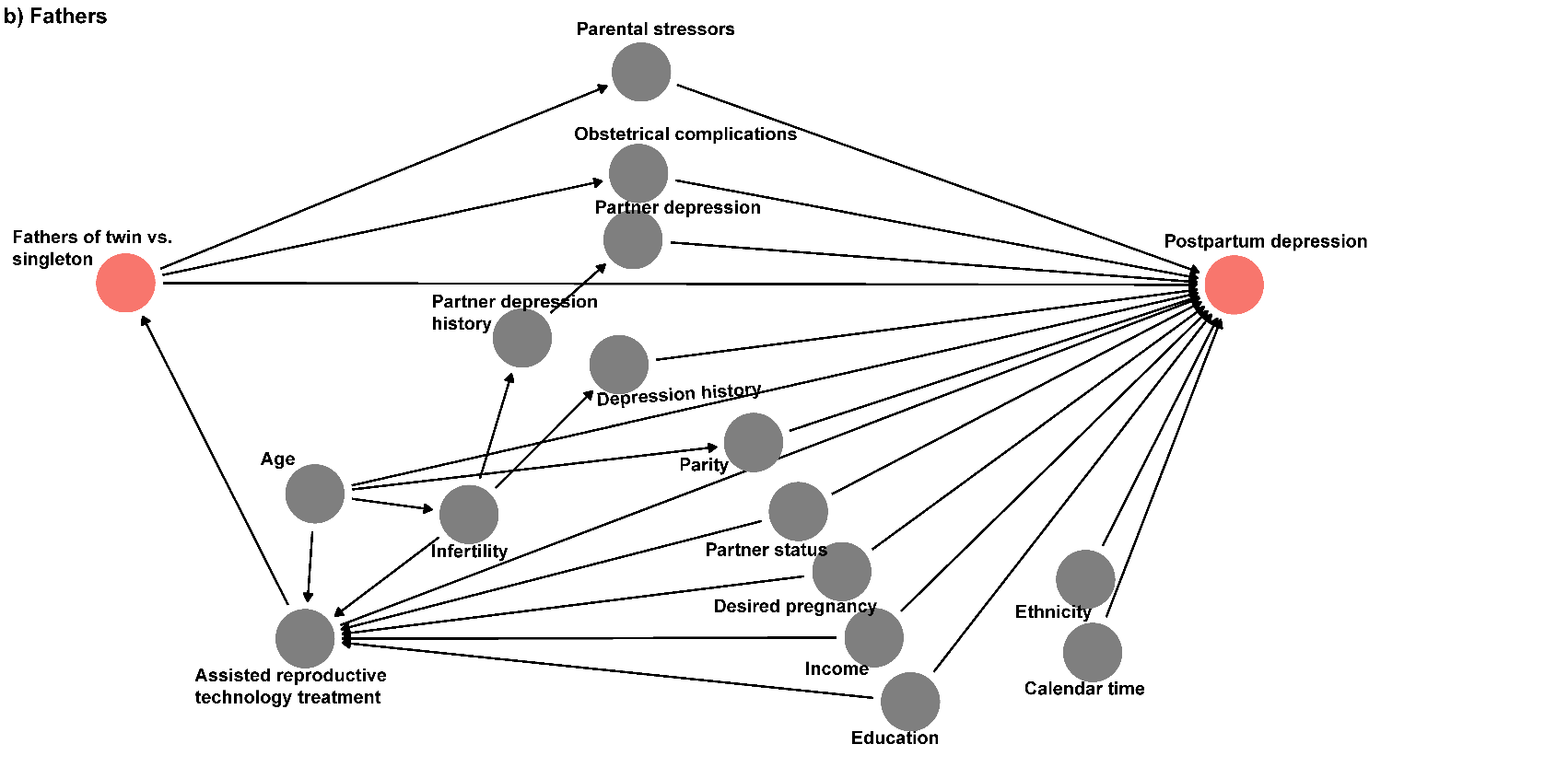
**

**Figure S2: Directed acyclic graph the association between a) mothers and b) fathers and risk of postpartum depression.**

1. **Depression diagnoses only**

**
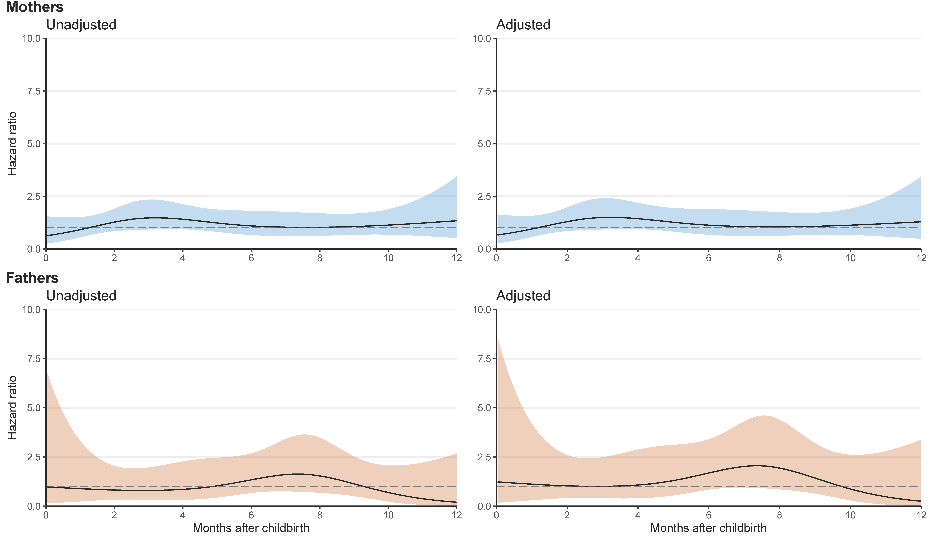
**

1. **Antidepressant prescriptions only**

**
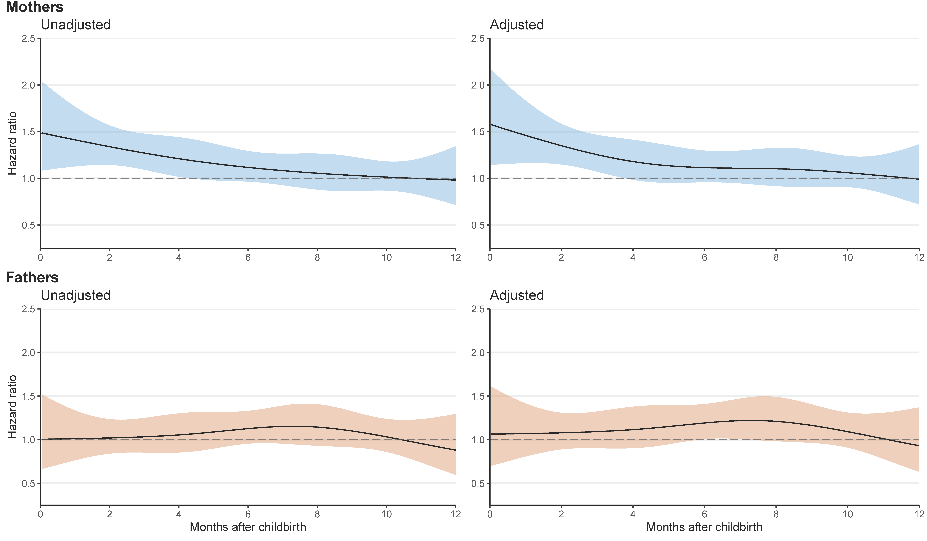
**

**c) Study period: 2007-2019**

**
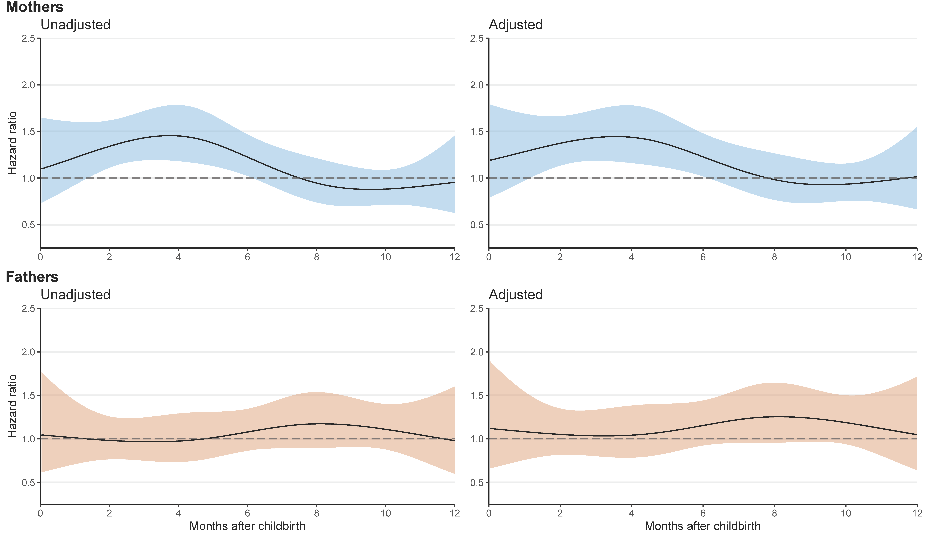
**

**Figure S3: Sensitivity analyses of hazard ratios of postpartum depression in twin compared to singleton mothers and fathers. Sensitivity analyses include a) restricting PPD to only depression diagnoses b) restricting PPD to only antidepressant prescriptions and c) restricting study period to only including parents of children born 2007-2019**

1. **Depression diagnoses only**

**
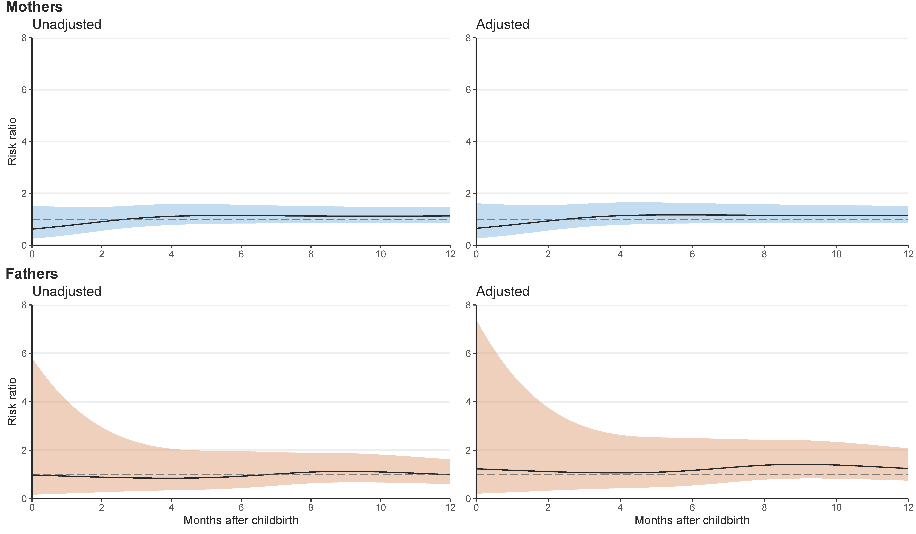
**

1. **Antidepressant prescriptions only**

**
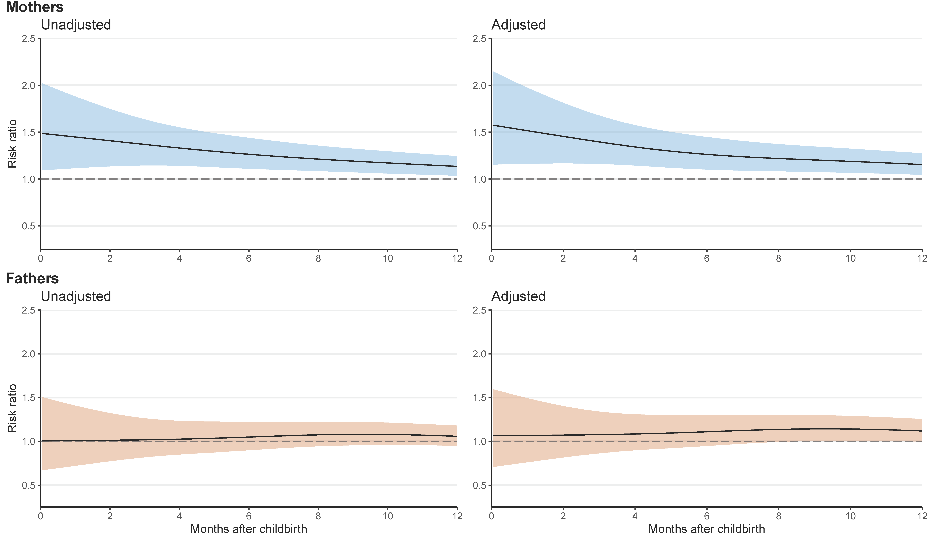
**

1. **Study period: 2007-2019**

**
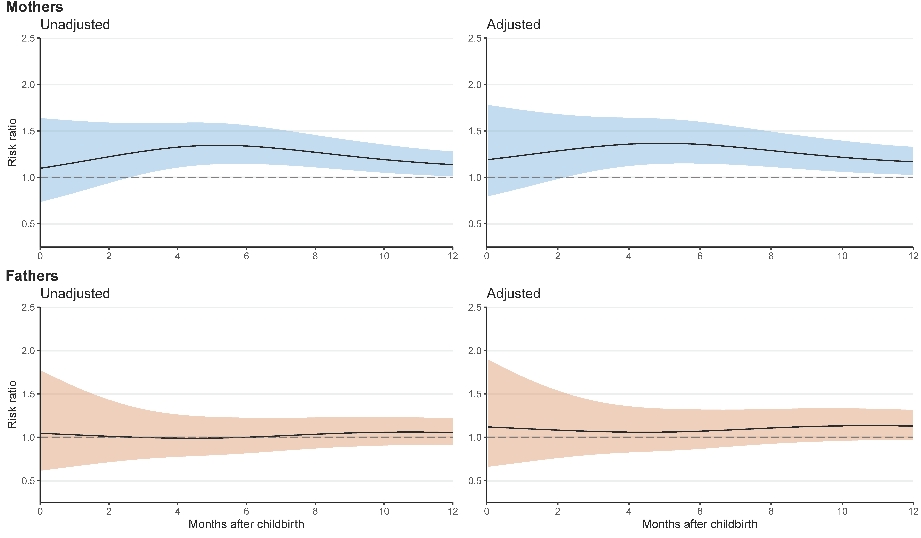
**

**Figure S4: Sensitivity analyses of hazard ratios of postpartum depression in twin compared to singleton mothers and fathers. Sensitivity analyses include a) restricting PPD to only depression diagnoses b) restricting PPD to only antidepressant prescriptions and c) restricting study period to only including parents of children born 2007-2019**
